# Synthesis and Biological Evaluation of Bisthiazoles and Polythiazoles

**DOI:** 10.3390/molecules23051133

**Published:** 2018-05-10

**Authors:** Mohammed A. Al-Omair, Abdelwahed R. Sayed, Magdy M. Youssef

**Affiliations:** 1 Department of Chemistry, Faculty of Science, King Faisal University, Hofuf 31982, Saudi Arabia; alomair@kfu.edu.sa (M.A.A.-O.); mmm_youssef@yahoo.com (M.M.Y.); 2 Department of Chemistry, Faculty of Science, Beni-Suef University, Beni-suef 62514, Egypt; 3 Department of Chemistry, Faculty of Science, Mansoura University, Mansoura 35516, Egypt

**Keywords:** hydrazonoyl, thiosemicarbazones, thiazoles, polythiazoles, DNA degradation, antioxidant, antimicrobial, IC_50_

## Abstract

Small heterocyclic compounds containing nitrogen and sulfur atoms, such as thiazole derivatives, represent a significant class of organic azoles that exhibit promising bioactivities and have a great potential in medicinal and agricultural fields. A convenient and high-yielding synthetic approach for a range of organic molecules is presented. The nuclease-like activities of compounds were studied with the aid of *E. coli* AB1157 DNA and agarose gel electrophoresis. The antioxidant evaluation of the compounds was carried out with different antioxidant techniques, such as ABTS and NO scavenging efficiency. The antibacterial behavior was evaluated against various bacterial strains, both Gram-positive and -negative, and the minimum inhibitory concentration (MIC) values of these compounds were determined. The antiproliferative activities and IC_50_ values of the synthesized organic molecules compounds against HEPG-2, MCF-7, and HCT-116 cell lines were evaluated.

## 1. Introduction

Hydrazonoyl halides are pivotal in chemical synthesis due to the fact that these compounds are synthetic precursors that generate a large number of alicyclic and organic molecules. Moreover, the hydrazonoyl halide derivatives exhibit significant biological activities, such as herbicidal, antimicrobial, acaricidal, antiviral, pesticidal, fungicidal, miticidal, antisarcoptic, insecticidal, anthelmintic, and antiarthropodal [1,2]. 

A series of thiosemicarbazone derivatives have shown to have a wide range of chemotherapeutic value and pharmacological importance. The bioactivities of thiosemicarbazone derivatives represent a wide range of properties including antitumor, cytotoxic [3], antibacterial [4], and antiviral [5] activities.

Thiazole derivatives are present in numerous pharmaceutically active compounds and play a significant role in agricultural industry. These have been reported to display antimicrobial [6,7], anti-rheumatoid [8], anti-hypertensive [9], anti-human immunodeficiency virus (HIV) [10], anticancer [11], antifever [12], anticonvulsant [13], herbicidal, insecticidal, schistosomicidal, and anthelmintic [14] activities. In addition, vitamin B1 also contains a thiazole ring in its structure, and this vitamin plays an essential role as coenzyme in the decarboxylation process of carbohydrate metabolism [15]. It is also required for diabetic patients to improve their carbohydrate catabolism. Moreover, a large number of thiazole-containing pharmaceutical compounds are present in several drug molecules, such as Tiazofurin (antineoplastic mediators), Bleomycin [16], Ritonavir (anti-HIV medication) [17], Fanetizole and Meloxicam (arthritis mediators) [18], Nizatidine (antiulcer mediator) [19], Imidacloprid (insecticide), and sulfathiazole as an antibiotic [20]. 

The thiazole motif is characterized as a ligand for estrogen [21] and adenosine [22] receptors. In addition, thiazole derivatives with diacylhydrazine substitutions at 2,4 positions have been reported as a PGE2 antagonist [23]. These derivatives have also been found to represent a new class of active molecules, which can be employed as therapeutic drugs in breast cancer and osteoporosis curing [24]. Literature reveals that the synthesis and reactivity of 5-keto thiazole to prepare diazonium chlorides is very simple [25]. Treatment of 1,3,4-oxadiazolyl with ethyl chloroacetate and α-haloketones affords thiazole derivatives [26]. Synthesis of 1,3-thiazoles via reaction of thiosemicarbazide derivatives with various halogens, such as ethyl bromoacetate and ethyl-2-bromopropionate, is also known [27]. Moreover, synthesis of bisthiazoles by treating dihydrazonoyl dichloride with hydrazone has also been documented [28,29].

The prime objective of this article relies on utilization of simple method to synthesize bioactive thiazoles and polythiazoles. The desired products have been evaluated using different biological assays such as antimicrobial, antioxidants, and nuclease-like activity assays and IC_50_ determination. 

## 2. Results and Discussion

### 2.1. Chemistry

**Scheme 1:**
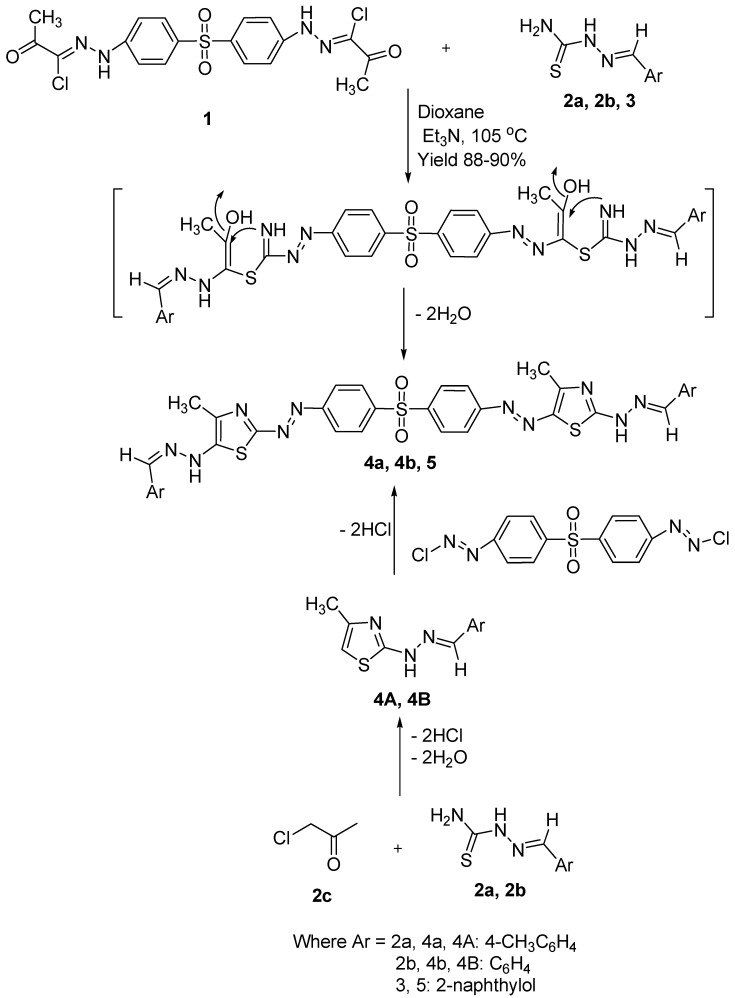
Synthesis of bisthiazoles **4a**, **4b**, and **5**.

We report here a rapid and simple method to synthesize thiazoles in excellent yield and short reaction time. We prepared thiazole derivatives **4a**, **4b**, and **5** using two different methods. The first method proceeded in two steps. The first step involved the preparation of thiosemicarbazones **2a**, **2b**, and **3** by reaction of aldehyde with thiosemicarbazide. In the second step, treatment of bishydrazonoyl **1** with 2-(arylidene)hydrazinecarbothioamides **2a**, **2b**, or **3** in dioxane and basic medium, under reflux for 30 min, proceeded through a nucleophilic substitution reaction followed by elimination of water to give thiazoles **4a**, **4b**, or **5** as the only isolated products (confirmed by thin layer chromatography) (Scheme 1). The reaction is rapid, simple, and gives excellent yields and has a short reaction time in comparison to previous method [28]. The obtained products were elucidated by spectral data. ^1^H NMR spectrum of thiazoles showed the absence of NH_2_ protons present in the starting material. The IR spectra revealed the absence of carbonyl absorption present in the starting bishydrazonoyl **1**, as shown in Scheme 1.

In the second method, an alternative method for the synthesis of bisthiazole **4a** and **4b**, took place by coupling compounds **4A**, **4B** [30] to aryldiazonium salts to furnish compounds **4a** and **4b**. These products showed similar IR spectra, m.ps., and mixed m.ps. to those from the reaction of **1** with **2a** or **2b**.

**Scheme 2:**
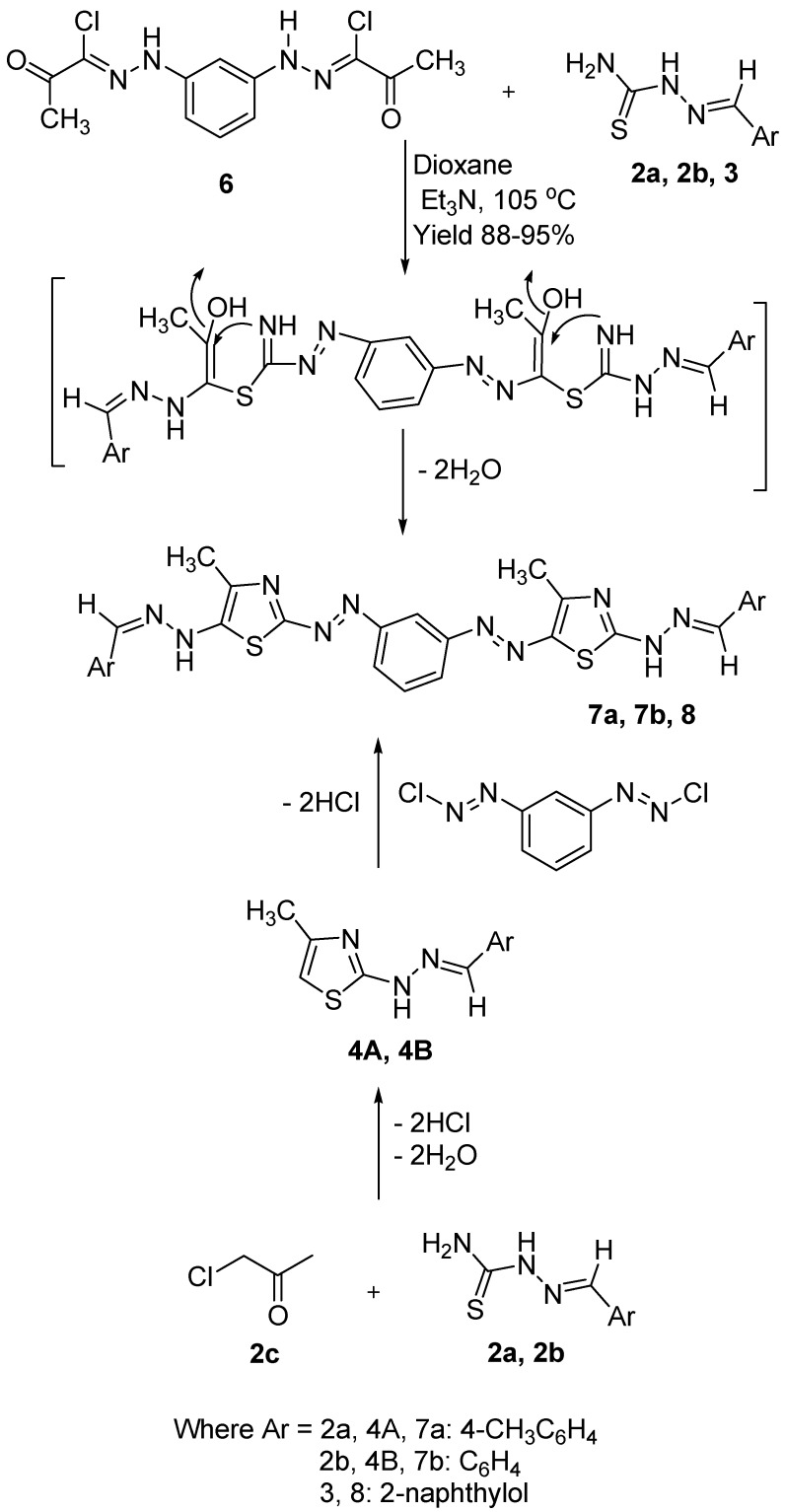
Synthesis of bisthiazoles **7a**, **7b**, and **8**.

Similarly, treatment of the bishydrazonoyl **6** with 2-(arylidene)hydrazinecarbothioamides **2a**, **2b**, and **3** in dioxane containing a catalytic amount of base and heating for 30 min furnished the desired bisthiazole derivatives **7a**, **7b**, and **8** in excellent yields and short reaction times (Scheme 2). It is envisaged that these compounds follow the same mechanistic route as that explained for the formation of **4a**, **4b**, and **5**. 

An alternative method for the synthesis of bisthiazoles **7a** and **7b** was performed based on the coupling of compounds **4A**, **4B** [30] to aryldiazonium salts to furnish compounds **7a** and **7b**. These products had identical IR spectra, m.ps., and mixed m.ps. when compared to the compounds obtained by the reaction of **6** with **2a** or **2b**.

**Scheme 3:**
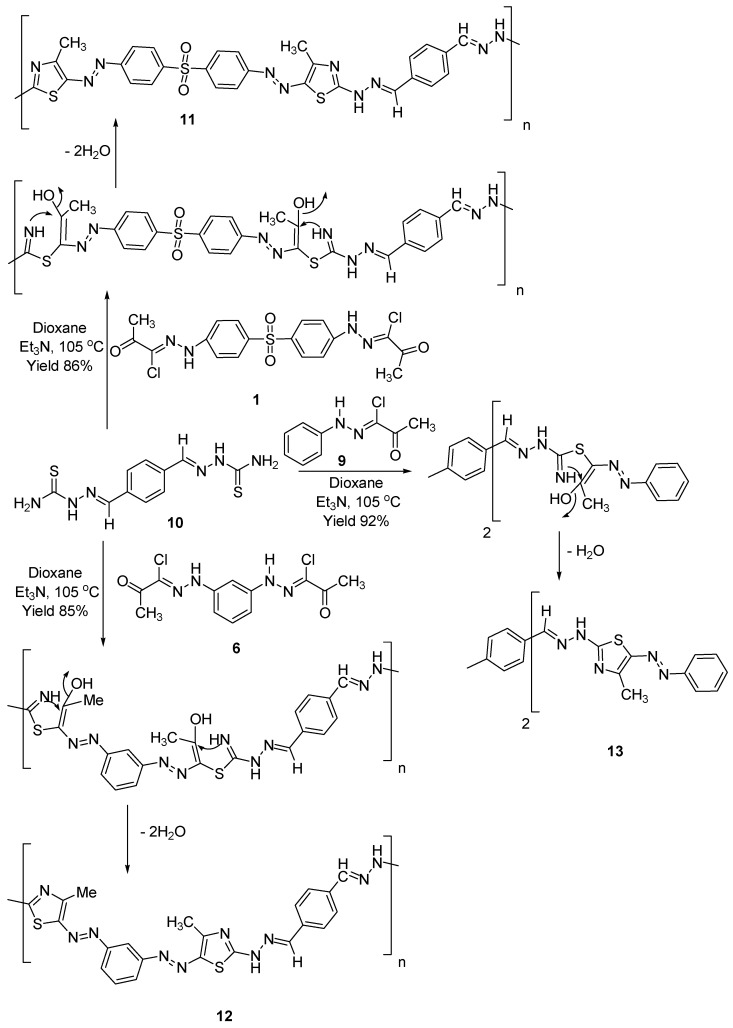
Synthesis of bisthiazole **13** and polythiazoles **11** and **12**.

In order to further explore this chemistry, 2,2′-(1,4-phenylenebis-(methanylylidene))-bis-(hydrazinecarbothioamide) **10** was reacted with monohydrazonoyl (α-halocarbonyl) **9** to give product **13**. We extended this work to prepare polythiazoles. Thus, the reaction of 2,2′-(1,4-phenylenebis(methanylylidene))bis-(hydrazinecarbothioamide) **10** with bishydrazonoyl halides containing α-halocarbonyl **1** or **6** gave products **11** and **12**, as described in Scheme 3. The mechanistic formation of the products is similar to that proposed for previous reactions, through elimination of hydrochloric acids to form S-alkylated intermediate, which cyclized by loss of water molecules. The IR spectra of the final products **11** and **12** showed absence of the carbonyl group band, which was present in the starting materials. In addition, the IR spectra of **11** and **12** revealed bands indicating a secondary amine stretch vibration at 3154 and 3364 cm^−1^ and a methyl group at 1356 and 1358 cm^−1^. The molecular weight of the polymers could not be analyzed due to poor solubility in different solvent. 

We checked the solubility of polythiazoles **11** and **12**. These polymers are soluble in *N*-methylpyrolidone (NMP), dimethylformamide (DMF), dimethylacetamide (DMA), and dimethylsulfoxide (DMSO). In addition, we found these compounds are insoluble in carbon tetrachloride, benzene, cyclohexane, methylene chloride, petroleum ether, chloroform, methanol, and ethanol.

### 2.2. Biological Evaluations

#### 2.2.1. DNA Digestion Pattern

The DNA digestion ability of the synthesized compounds **1**, **4a**, **4b**, **5**, **6**, **7a**, **7b**, **8**, **9**, **11**, **12**, and **13** compared to that of controls is owing to their DNA affinity and digestion ability. The synthesized compounds were permitted to bind with DNA extracted from *E. coli* AB1157 for studying the DNA digestion ability. In the current study, the compounds exhibited an increase in the DNA digestion ability.

**Figure 1:**
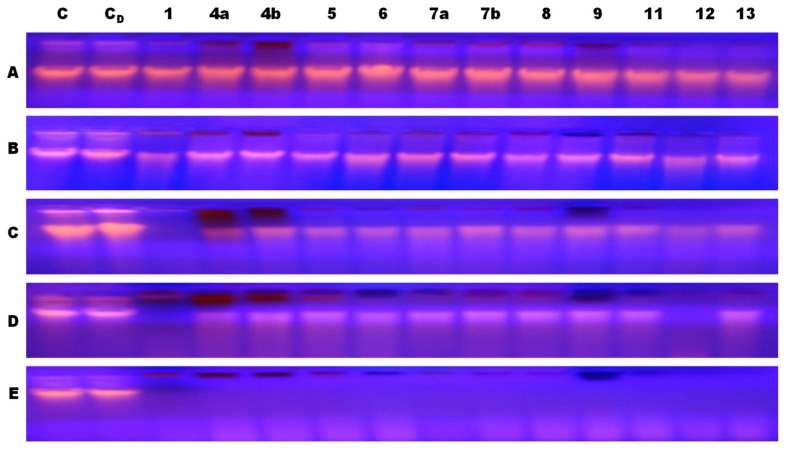
A figure showing the degradation effect of 2 (**A**), 4 (**B**), 6 (**C**), 8 (**D**), and 10 (**E**) µM of compounds (**1**, **4a**, **4b**, **5**, **6**, **7a**, **7b**, **8**, **9**, **11**, **12**, and **13**) (Lanes 3–14) on the genomic DNA isolated from *E. coli*. Lane 1 *E. coli* DNA; Lane 2 *E. coli* DNA + DMSO.

By varying the concentration of the tested compounds, their ability to interact with the DNA was demonstrated, as represented in Figure 1. DNA digestion activity of the compounds was examined in aerobic conditions at 37 °C and in the absence of any exterior additives. The results verified that the synthesized compounds show promising ability to bind and digest the DNA. The achieved results show that the synthesized compound **1** at concentration 4 µg has nuclease-like activities, as the DNA has a smear, as represented in Figure 1B, Lane 3. Moreover, compound **1** at concentration 6 µg has higher DNA affinity and accomplished DNA degradation, as shown in Figure 1C,D, whereas at concentration of 2 µg, compound **1** displayed no cleavage effect on the genomic DNA compared to the controls (Figure 1A). After incubation for 1 h, the DNA was substantially digested at a concentration of 6 µg by compounds **4a**, **6**, **7a**, **7b**, **8**, **11**, **12**, and **13**, while at 6 µg of compound **1**, the DNA was entirely cleaved, as shown in Figure 1B,C. Instead, the smeared DNA detected with compounds **4b**, **5**, and **9** at 8 µg may be due to their cytostatic binding ability. The genomic DNA was completely degraded by the compounds at concentration 10 µg, as represented in Figure 1E. These data indicate that the DNA cleaving activity of **1**, **4a**, **4b**, **5**, **6**, **7a**, **7b**, **8**, **9**, **11**, **12**, and **13** depend on the concentration and the structure of the studied samples. The increase in nuclease-like activities of compound **1** may be due to the electron withdrawing activity of the sulfonyl moiety and the negative inductive effect of two Cl atoms, which lie between C=O and =N–N–H as electron donating groups. The results of DNA degradation by the tested compounds was in agreement with that formerly stated [31,32,33,34,35]. In conclusion, the present study proves that the compounds have substantial DNA digestion abilities, in the absence of external materials, at 10 µg. The DNA cleavage activity, deprived of any additional material, is valuable information that renders the synthesized compounds promising agents for antitumor treatment.

#### 2.2.2. Antioxidant Activities

Subsequent to the DNA digestion investigation showing that the synthesized organic molecules displayed respectable binding and affinity toward DNA at concentration of 10 µg, the activity of the organic molecules as antioxidants was examined. The ABTS test is broadly utilized for measuring the scavenging activity ability of the ABTS^•+^ radical. Typically, existing as a moderately stable molecule, the ABTS free radical cation displays a sharp absorption band in the visible spectrum at 734 nm. The antioxidant efficiency of the tested compounds was assessed and the scavenging effect of ABTS cation by the compounds were in the following order: **1** > **12** > **4a** > **6** > **11** > **13**, as illustrated in Table 1, while organic molecules **4b**, **5**, **7a**, **7b**, **8**, and **9** displayed good activity. Compounds **1**, **4b**, **6**, **8**, **12**, **11**, and **13** exhibited better activity compared to the other studied compounds. The presence of 4-CH_3_C_6_H_4_ in the structure of thiazole compound **4a** increased the antioxidant activity more than that with C_6_H_4_ in the structure of thiazole compound **4b**.

**Figure 2:**
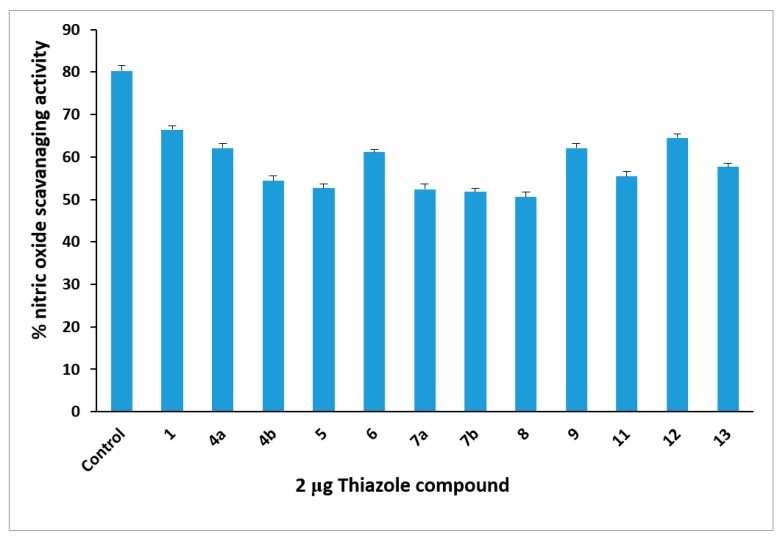
The nitric oxide scavenging activity of the tested compounds.

The organic molecules were similarly established for their antioxidant activities by nitric oxide (NO) scavenging action evaluation method (Figure 2). The compounds **1**, **4a**, **6**, **9**, **11**, **12**, and **13** showed good radical scavenging activity. Other thiazole compounds, **5** and **8**, showed significant antioxidant efficiency, while the thiazoles **7a** and **7b** exhibited moderate NO scavenging compared with the control. The ability of the compounds to respond to the development of nitric oxide indicates the possibility for these compounds to mitigate harm caused by excess NO in vivo. The present work obviously proves that organic molecules **1**, **4a**, **6**, **9**, **11**, **12**, and **13** are powerful NO scavengers. Nitric oxide has been documented to play an important and useful role in a variety of biochemical metabolic pathways. NO is a small free radical formed non-enzymatically and causes harm to most high molecular weight molecules, such as protein, lipids, and DNA. It also exacerbates cancer, swelling, and other injurious situations [31]. NO produced from sodium nitroprusside reacts with O_2_ to generate NO_2_. Therefore, the compounds may prevent NO_2_ generation by interacting with O_2_ to prevent its reaction with NO. The existence of C_6_H_4_–CH=N–NH– in the compounds increases its antioxidant efficiency.

**Figure 3:**
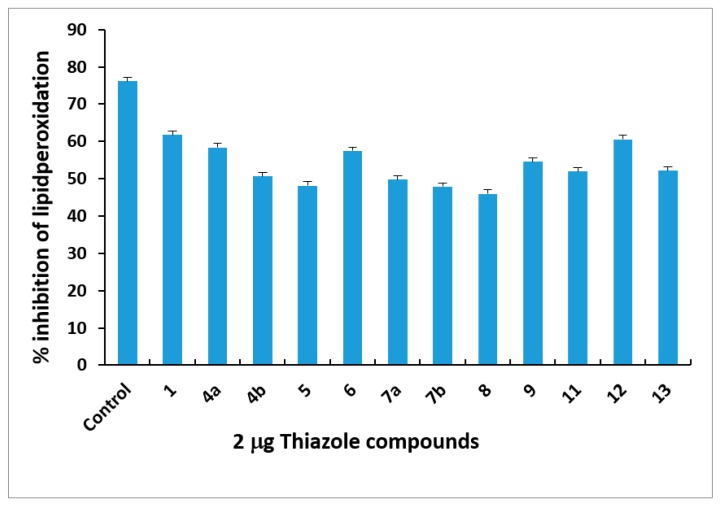
The activity of the tested compounds against lipid peroxidation.

The occurrence of antioxidant activities in the tested organic molecules causes the reduction of the ferricyanide/Fe^3+^ composite to the Fe^++^ oxidation state. Consequently, ferrous reduction can be observed by measuring blue Perl’s Prussian at 700 nm. The creamy color of the test solution changes to different shades of blue and green, depending on the antioxidant power of the organic molecules. The antioxidant power of the compounds may act as an important proxy of its antioxidant potential efficiency. As revealed by Figure 3, the thiazole compounds had a powerful and effective antioxidant effect on the ferricyanide/Fe^3+^ composite when compared to the control. For the determination of the antioxidant ability of the compounds, the Fe^2+^/Fe^3+^ oxidation state was examined in the presence of the compounds. The resulting antioxidant activities demonstrate the electron-donating capability of the compounds, thus scavenging free radicals and creating stable products. The consequence of the oxidation–reduction response is the elimination of free radical chain responses that may be harmful. The compounds displayed an effective antioxidant potential in the fatty acid emulsification structure. The result of 2 μg of the compounds on the peroxidation of fatty acid (linoleic) emulsification is presented in Figure 3. The linoleic acid autoxidation in emulsion without compounds or control resulted in a quick increase in peroxides. Therefore, these results obviously indicate that the compounds **1**, **4a**, **6**, **9**, **11**, and **12** had sufficient antioxidant efficiency compared to the control. The presence of Cl atoms in the structure of compound **6** increase the antioxidant properties compared to that recorded in the absence of Cl atoms in the structure of compounds **11** and **13**.

#### 2.2.3. Antibacterial Activities

The antibacterial activities of compounds **1**, **4a**, **4b**, **5**, **6**, **7a**, **7b**, **8**, **9**, **11**, **12**, and **13** were verified against both Gram-positive and Gram-negative strains, using only one concentration from them in DMSO as a control. The diameters of inhibition zones resulting from the tested compounds applied to the established bacterial strains are shown in Table 2. From the data, it is clear that the tested organic molecules **1**, **4a**, **6**, **11**, and **12** exhibit the strongest antimicrobial activities towards all the investigated microorganisms compared to the ampicillin as a reference antibiotic. The tested compounds **4b**, **5**, **7a**, **7b**, and **13** reveal good antimicrobial activity against the surveyed microorganisms. However, compound **8** revealed the lowest antimicrobial activity in this work. 

Based on the previously established structures of organic molecules’ and their activity against bacterial strains, it is apparent that for the compounds **1**, **4a**, **6**, **11**, and **12**, the presence of electron donating groups CH_3_, S, N, and aromaticity, respectively, increase the antibacterial activities of the tested compounds. This may be owing to the existence of N and S atoms in the tested compound. It has been proposed that the compounds that have N and S atoms might repress enzyme activity, since enzymes which require certain clusters for their activity are especially susceptible to deactivation by the tested compounds [32].

The minimum inhibitory concentration (MIC) of the synthesized compounds **1**, **4a**, **4b**, **5**, **6**, **7a**, **7b**, **8**, **9**, **11**, **12**, and **13** is defined as the concentration at which no bacterial growth was detected. The MIC of the compounds toward *E. coli*, *P. aeruginosa*, *B. megaterium*, and *S. aureus* bacterial strains was evaluated. The MIC values (in μg/mL) of the tested compounds toward the above bacterial strains is presented in Table 3. The compounds **1**, **4a**, **6**, **11**, **12**, and **13** exhibited extreme inhibitory activities towards, *E. coli*, *P. aeruginosa*, *S. aureus*, and *B. megaterium* bacterial strains (MIC standards in the range of 30–45 μg/mL). The compounds **4b**, **5**, **7a**, **7b**, and **9** had moderate inhibitory activities towards all tested bacterial strains (MIC range: 50–70 μg/mL). A reasonable conclusion based on the obtained effects is that the antibacterial activities of the compounds is caused by their basic assembly and, furthermore, by the presence of S and N atoms, phenyl ring, and CH_3_ and Cl groups. 

#### 2.2.4. Cytotoxicity Evaluation

In the current study, the cytotoxicity assay was performed utilizing hepatocellular carcinoma (HEPG-2), mammary gland (MCF-7), and colorectal carcinoma (HCT-116) cells (Table 4) to evaluate the anti-proliferative activities of the synthesized organic molecules (µM). Doxorubicin was utilized as a standard anticancer agent, which has very strong anticancer activities. Cytotoxicity was stated as the concentration that produced 50% loss (IC_50_) of the cell monolayer. As illustrated Table 4, compound **1** has very strong anticancer activities against cell lines HePG-2, HCT-116, and MCF-7, with IC_50_ values of 8.92 ± 0.6, 8.22 ± 0.9, and 6.89 ± 0.4 μM, respectively. The thiazole compound **4a** also exhibited very strong anticancer activities against all the studied cell lines (HePG-2, HCT-116, and MCF-7) with IC_50_ values 9.94 ± 0.5, 8.87 ± 0.7, and 7.83 ± 0.6 μM, respectively. The thiazole compounds **6**, **11**, and **12** displayed strong anticancer activities against HePG-2, HCT-116, and MCF-7 cell lines, as represented in Table 4. The compounds **4b**, **5**, **7a**, **7b**, and **13** revealed moderate anticancer activities against all tested cell lines. Compounds **8** and **9** presented weak anticancer activities against all tested cell lines. The presence of two chlorine atoms and sulfonyl moiety (negative inductive effect, electron withdrawing group) in compound **1** increases the cytotoxicity activity towards HePG-2, HCT-116, and MCF-7. The thiazole moiety in compounds **4a**, **6**, **11**, and **12** enhanced the cytotoxicity activity towards HePG-2, HCT-116, and MCF-7 cell lines. In compounds **11** and **12**, the presence of polythiazole moiety increased the cytotoxicity activity against the studied cell lines.

## 3. Experimental

### 3.1. Synthesis

All melting points were determined on an electrothermal apparatus and are uncorrected. IR spectra were recorded (KBr discs) on a Shimadzu FT-IR 8201 PC spectrophotometer (Shimadzu, Tokyo, Japan). ^1^H NMR spectra were recorded in CDCl_3_ and (CD_3_)_2_SO solutions on a Varian Gemini 300 MHz spectrometer (Agilent, Palo Alto, CA, USA), and chemical shifts are expressed in δ units using TMS as an internal reference. Mass spectra were recorded on a Shimadzu GC-MS QP 1000 EX instrument. Elemental analyses were carried out at the Microanalytical Canter of Cairo University. 

Synthesis of thiazoles (**4a**, **4b**, **5**, **7a**, **7b**, **8**, **13**) and polythiazoles (**11** and **12**):

**Method A:** To equivalent mole of **1**, **6**, or **9** in dioxane, an equivalent mole from thiosemicarbazones **2a**, **2b**, **3**, or **10** was added in the presence of an equivalent mole of triethylamine. The reaction mixtures were heated for 30 min and then allowed to cool. The solid formed was filtered off and then recrystallized from dimethylformamide/methanol to get thiazoles **4a**, **4b**, **5**, **7a**, **7b**, **8**, **13**, and polythiazoles **11** and **12**.

**Method B:** (alternate synthesis of **4a**, **4b**, **7a**, and **7b**): Diazotized diamine, synthesized by diazotizing the diamine (5 mmol), dissolved in hydrochloric acid (6 M, 6 mL) with sodium nitrite (0.71 g, 10 mmol) in water (5 mL), was added dropwise to a stirred solution of **4A** [30] or **4B** [30] (10 mmol) in pyridine (40 mL) at 0–5 °C. The reaction mixture was chilled in an ice bath at 0–5 °C for 2 h with strong stirring and then left in refrigerator for 24 h. The final precipitated solid was collected and washed several times with water, dried and crystallized from dimethylformamide/methanol to afford the final products **4a**, **4b**, **7a**, and **7b**. 

*5,5′-(4,4′-Diphenylsulphone-4,4′-diyl)-bis-({2-(4-methylbenzylidenehydrazino}-4-methyl-5-azo-1,3-thiazole)* (**4a**) [28]. Brown-red solid; Yield (90%); mp 248–250 °C (DMF/MeOH). IR (cm^−1^) (KBr): ν_max_ 3220 (NH), 1599 (C=C), 1549 (N=N), 1378 (CH_3_) cm^−1^. ^1^H NMR (DMSO-*d*_6_): 2. 39 (s, 6H, 2CH_3_), 2.58 (s, 6H, 2CH_3_), 7.13–7.79 (m, 16H, ArH’s), 8.59 (s, 2H, N=CH) and 10.95 (s, 2H, NH), ^13^C NMR (DMSO-*d*_6_): at 16.8, 21.6, 115.2, 129.4, 130.0, 130.4, 132.4, 136.2, 140.0, 141.9, 142.0, 143.2, 160.1 and 171.8 ppm. MS *m*/*z* (%): 732 (M^+^, 63). Analysis Calcd for C_36_H_32_N_10_O_2_S_2_ (732.19): C, 59.00%; H, 4.40%; N, 19.11%; Found: C, 59.01%; H, 4.36%; N, 19.09%. 

*5,5′-(4,4′-Diphenylsulphone-4,4′-diyl)-bis-({2-(benzylidenehydrazino}-4-methyl-5-azo-1,3-thiazole)* (**4b**) [28]. Red solid; Yield (88%); mp 256–259 °C (DMF/MeOH). IR (cm^−1^) (KBr): ν_max_ 3226 (NH), 1598 (C=C), 1549 (N=N), 1377 (CH_3_) cm^−1^. ^1^H NMR (DMSO-*d*_6_): 2.58 (s, 6H, 2CH_3_), 7.46–7.76 (m, 18H, ArH’s), 8.71 (s, 2H, N=CH) and 10.92 (s, 2H, NH), ^13^C NMR (DMSO-*d*_6_): at 16.6, 114.4, 128.4, 128.9, 128.9, 131.8, 133.7, 134.4, 140.9, 147.4, 160.8, 172.6 and 178.9. MS *m*/*z* (%): 704 (M^+^, 79). Analysis Calcd for C_34_H_28_N_10_O_2_S_3_ (704.16): C, 57.94%; H, 4.00%; N, 19.87%; Found: C, 57.89%; H, 3.99%; N, 19.81%%.

*5,5′-(4,4′-Diphenylsulphone-4,4′-diyl)-bis-({2-(2-hydroxynaphthylidenehydrazino}-4-methyl-5-azo-1,3-thiazole)* (**5**). Black-brown solid; Yield (90%); mp > 300 °C (DMF/MeOH). IR (cm^−1^) (KBr): ν_max_ 3425 (OH), 3213 (NH), 1598 (C=C), 1551 (N=N), 1379 (CH_3_) cm^−1^. ^1^H NMR (DMSO-*d*_6_): 2.56 (s, 6H, 2CH_3_), 7.03–7.97 (m, 20H, ArH’s), 8.84 (s, 2H, N=CH), 10.73 (s, 2H, OH) and 10.99 (s, 2H, NH), ^13^C NMR (DMSO-*d*_6_): at: 16.5, 114.8, 126.9, 127.9, 128.2, 128.5, 129.4, 131.7, 132.9, 136.3, 140.1, 142.3, 144.7, 146.9, 152.4, 162.1 and 171.4 ppm. MS *m*/*z* (%): 836 (M^+^, 72). Analysis Calcd for C_42_H_32_N_10_O_4_S_3_ (836.18): C, 60.27%; H, 3.85%; N, 16.74%; Found: C, 60.25%; H, 3.82%; N, 16.76%.

*5,5′-(Phenyl-1,3-diyl)-bis-({2-(4-methylbenzylidenehydrazino}-4-methyl-5-azo-1,3-thiazole)* (**7a**) [28]. Red crystal solid; Yield (88%); mp 181–183 °C (DMF/MeOH). IR (cm^−1^) (KBr): ν_max_ 3157 (NH), 1592 (C=C), 1547 (N=N), 1367 (CH_3_) cm^−1^. ^1^H NMR (DMSO-*d*_6_): 2.38 (s, 6H, 2CH_3_), 2.54 (s, 6H, 2CH_3_), 7.01–7.88 (m, 12H, ArH’s), 8.45 (s, 2H, N=CH) and 10.71 (s, 2H, NH), ^13^C NMR (DMSO-*d*_6_): at 18.0, 21.6, 113.8, 125.9, 126.8, 127.4, 129.3, 130.4, 132.1, 133.9, 144.1, 150.1, 160.7 and 167.1 ppm. MS *m*/*z* (%): 592 (M^+^, 76). Analysis Calcd for C_30_H_28_N_10_S_2_ (592.74): C, 60.79%; H, 4.76%; N, 23.63%; Found: C, 60.73%; H, 4.77%; N, 23.59%.

*5,5′-(Phenyl-1,3-diyl)-bis-({2-(benzylidenehydrazino}-4-methyl-5-azo-1,3-thiazole)* (**7b**) [28]. Red solid; Yield (91%); mp 201 °C (DMF/MeOH). IR (cm^−1^) (KBr): ν_max_ 3167 (NH), 1609 (C=N), 1542 (N=N), 1362 (CH_3_) cm^−1^. ^1^H NMR (DMSO-*d*_6_): 2.5 (s, 6H, 2CH_3_) 7.03–7.8 (m, 14H, ArH’s), 8.5 (s, 2H, N=CH) and 10.8 (s, 2H, NH), ^13^C NMR (DMSO-*d*_6_): at 20.9, 112.1, 115.2, 125.8, 126.9, 128.5, 130.0, 130.6, 131.1, 138.2, 139.3, 149.5 and 166.6 ppm; *m*/*z* (%): 564 (M^+^, 32). Analysis Calcd for C_28_H_24_N_10_S_2_ (564.69): C, 59.56%; H, 4.28%; N, 24.80%; Found: C, 59.51%; H, 4.25%; N, 24.81%.

*5,5′-(Phenyl-1,3-diyl)-bis-({2-(2-hydroxynaphthylidenehydrazino}-4-methyl-5-azo-1,3-thiazole)* (**8**). Black-brown crystal solid; Yield (95%); mp > 300 °C (DMF/MeOH). IR (KBr): ν_max_ 3435 (OH), 3179 (NH), 1610 (C=N), 1549 (N=N), 1371 (CH_3_) cm^−1^. ^1^H NMR (DMSO-*d*_6_): 2.63 (s, 6H, 2CH_3_) 7.09–8.01 (m, 16H, ArH’s), 8.79 (s, 2H, N=CH), 10.79 (s, 2H, OH) and 10.92 (s, 2H, NH), ^13^C NMR (DMSO-*d*_6_): at: 20.9, 113.4, 117.1, 126.3, 127.9, 128.4, 130.2, 130.9, 131.2, 132.3, 135.9, 136.4, 137.5, 142.5, 149.1, 159.8 and 166.9 ppm. MS *m*/*z* (%): 696 (M^+^, 72). Analysis Calcd for C_36_H_28_N_10_O_2_S_2_ (696.18): C, 62.05%; H, 4.05%; N, 20.10%; Found: C, 60.01; H, 4.02; N, 20.11%.

*Poly(5{4,4′-diphenylsulphone-4,4′-diyl}({2-(benzylidenehydrazino}-4-methyl-5-azo-1,3-thiazole*) (**11**). Deep red-brown crystal solid; Yield (86%); mp > 300 °C (DMF/MeOH). IR (cm^−1^) (KBr): ν_max_ 3364 (NH) 1589 (C=C), 1554 (N=N), 1356 (CH_3_) cm^−1^. ^1^H NMR (DMSO-*d*_6_): 2. 36 (s, CH_3_), 2.59 (s, CH_3_), 7.08–8.01 (m, ArH’s), 8.63 (s, N=CH) and 10.72 (s, NH). ^13^C NMR and molecular weight of the polymers could not be recorded due to poor solubility of isolated products in different solvent. 

*Poly(5-{Phenyl-1,3-diyl}-2-{benzylidenehydrazino}-4-methyl-5-azo-1,3-thiazole*) (**12**). Deep red-brown crystal solid; Yield (85%); mp > 300 °C (DMF/MeOH). IR (cm^−1^) (KBr): ν_max_ 3154 (NH), 1604 (C=C), 1552 (N=N), 1358 (CH_3_) cm^−1^. ^1^H NMR (DMSO-*d*_6_): 2.35 (s, CH_3_), 2.58 (s, CH_3_), 6.99–7.89 (m, ArH’s), 8.57 (s, N=CH) and 10.66 (s, NH). ^13^C NMR and molecular weight of the polymers could not be recorded due to poor solubility of isolated products in different solvent. 

*1,4-Bis(2-{4-methyl-5-[phenyldiazenyl]thiazol-2-yl]hydrazono}methyl)benzene* (**13**). Red crystal solid; Yield (92%); mp 241–243 °C (DMF/MeOH). IR (cm^−1^) (KBr): ν_max_ 3241 (NH), 1597 (C=N), 1358 (CH_3_) cm^−1^. ^1^H NMR (DMSO-*d*_6_): 2.63 (s, 6H, 2CH_3_), 7.01–7.83 (m, 14H, ArH’s), 8.71 (s, 2H, N=CH) and 10.71 (s, 2H, NH), ^13^C NMR (DMSO-*d*_6_): at 16.5, 111.2, 128.0, 128.1, 128.8, 131.5, 133.8, 134.7, 140.9, 147.2 and 174.2ppm. MS *m*/*z* (%): 565 (M^+^, 90). Analysis Calcd for C_28_H_24_N_10_S_2_ (564.16): C, 59.56%; H, 4.28%; N, 24.80%; Found: C, 59.52%; H, 4.30%; N, 24.78%.

### 3.2. Biological Evaluations

#### 3.2.1. DNA Digestion Pattern

The DNA digestion pattern of the synthesized thiazoles was performed according to the reported procedure [32].

#### 3.2.2. ABTS Antioxidant Assay

The ABTS antioxidant assay was carried out according to the reported method [33].

#### 3.2.3. NO Scavenging Method

Griess reagent [31] was made and the NO scavenging method was performed according to the reported procedure [32].

#### 3.2.4. Determination of Total Antioxidant Activity in Linoleic Acid Emulsion

The total antioxidant activity of the compounds was determined according to the Fe^+2^ to Fe^+3^ reported procedure [31,32,33,34,35]. 

#### 3.2.5. Antibacterial Activities

The antimicrobial evaluation of the tested compounds was performed via cup diffusion procedure [36]. The Luria–Bertani agar medium was made as described [37]. 

#### 3.2.6. Minimum Inhibitory Concentrations (MIC)

Evaluation the MIC of the tested organic molecules was made according to the standard procedures as described by CLSI/NCCLS methods [38]. 

#### 3.2.7. Cytotoxicity Assay

The cell lines HEPG-2, MCF-7, and HCT-116 were obtained from ATCC via holding company for biological products and vaccines (VACSERA), Cairo, Egypt. Doxorubicin was used as a standard anticancer drug for comparison. The cell lines stated above were utilized to evaluate the inhibitory effects of the thiazole derivatives according to the reported method [39].

## 4. Conclusions

We report here a rapid and simple synthesis of bioactive bisthiazoles and polythiazoles in excellent yields and short reaction times. The desired products have been fully characterized. The nuclease-like activities of organic molecules were studied with the aid of *E. coli* AB1157 DNA and agarose gel electrophoresis. The antioxidant evaluation of the compounds was carried out with different antioxidant techniques, including ABTS and NO scavenging efficiency. The antibacterial behavior was evaluated against various bacterial strains of Gram-positive and -negative and the MIC values of these compounds was determined. The antiproliferative activities and IC_50_ of the synthesized compounds against HEPG-2, MCF-7, and HCT-116 cell lines was evaluated.

## Figures and Tables

**Table 1:** Antioxidant activity assay (ABTS) of the tested compounds.

Compound No.	% Inhibition
Control of ABTS	-
Ascorbic acid	86.2
**1**	67.4
**4a**	61.2
**4b**	53.7
**5**	51.9
**6**	60.1
**7a**	51.2
**7b**	50.3
**8**	48.1
**9**	49.4
**11**	58.5
**12**	63.7
**13**	56.1

**Table 2:** Effect of novel compounds on Gram-negative and Gram-positive microorganisms. The results are expressed as zone inhibition in mm diameter.

Compound	Gram-Negative		Gram-Positive	
	*E. coli*	*P. aeruginosa*	*S. aureus*	*B. megaterium*
**1**	17	16	17	16
**4a**	16	15	16	16
**4b**	12	11	14	12
**5**	12	12	11	10
**6**	15	15	14	15
**7a**	13	12	13	12
**7b**	12	12	11	11
**8**	10	10	11	11
**9**	15	15	14	14
**11**	15	14	17	12
**12**	16	15	16	14
**13**	15	14	13	14
**Ampicillin**	19	20	23	21

**Table 3:** Minimum inhibitory concentration (MIC) of novel compounds (µg/mL) against four microorganisms.

Compound	*E. coli*	*P. aeruginosa*	*B. megaterium*	*S. aureus*
**Ampicillin**	20	25	25	15
**1**	35	35	40	35
**4a**	35	35	35	40
**4b**	55	50	65	60
**5**	50	55	60	65
**6**	40	45	45	40
**7a**	60	65	70	70
**7b**	65	55	65	65
**8**	75	70	80	80
**9**	60	70	65	70
**11**	40	45	40	40
**12**	34	40	45	40
**13**	40	45	40	45

**Table 4:** Cytotoxic activity of derivatives (µM) against human tumor cells.

Compounds	In Vitro Cytotoxicity IC_50_ (µM) •		
	HePG-2	HCT-116	MCF-7
**DOX ••**	4.50 ± 0.2	5.23 ± 0.3	4.17 ± 0.2
**1**	8.92 ± 0.6	8.22 ± 0.9	6.89 ± 0.4
**4a**	9.94 ± 0.5	8.87 ± 0.7	7.83 ± 0.6
**4b**	25.49 ± 1.7	21.43 ± 1.5	37.64 ± 1.2
**5**	24.48 ± 1.3	33.24 ± 2.2	23.47 ± 2.0
**6**	10.41 ± 1.1	12.66 ± 1.3	16.57 ± 1.5
**7a**	36.57 ± 3.2	48.52 ± 2.9	26.13 ± 2.8
**7b**	35.53 ± 2.6	24.43 ± 1.7	27.92 ± 1.6
**8**	53.54 ± 2.1	61.41 ± 2.8	69.33 ± 2.6
**9**	83.56 ± 4.1	71.13 ± 3.6	53.91 ± 3.5
**11**	18.52 ± 1.3	14.72 ± 1.1	17.90 ± 1.4
**12**	11.25 ± 1.4	19.54 ± 1.7	12.66 ± 1.5
**13**	30.75 ± 2.3	41.37 ± 2.5	35.11 ± 2.4

**•** IC_50_ (µM): 1–10 (very strong), 11–20 (strong), 21–50 (moderate), 51–100 (weak), and above 100 (non-cytotoxic); **•• DOX**: Doxorubicin.
